# Best Nursing Intervention Practices to Prevent Non-Communicable Disease: A Systematic Review

**DOI:** 10.3389/phrs.2022.1604429

**Published:** 2022-09-14

**Authors:** Mercedes Gomez del Pulgar, Miguel Angel Cuevas-Budhart, Sonsoles Hernández-Iglesias, Maria Kappes, Veronica Andrea Riquelme Contreras, Esther Rodriguez-Lopez, Alina Maria De Almeida Souza, Maximo A. Gonzalez Jurado, Almudena Crespo Cañizares

**Affiliations:** ^1^ Centro de Educación Superior Hygiea, Madrid, Spain; ^2^ Coordination of the Center for Advanced Clinical Simulation of the Nursing Degree, Universidad Francisco de Vitoria, Posuelo de Alarcon, Madrid, España; ^3^ Unidad de Investigación Médica en Enfermedades Nefrológicas, Instituto Mexicano del Seguro Social (IMSS), Mexico City, México; ^4^ Institutional Relations and Health Practices of Health Sciences, Faculty of the Nursing Degree, Universidad Francisco de Vitoria, Pozuelo de Alarcon, Madrid, España; ^5^ College of Health Care Sciences, Nursing School, Universidad San Sebastián, Puerto Montt, Chile; ^6^ San Sebastián University, Santiago, Chile; ^7^ Spanish Nursing Research Institute, Consejo General de Enfermería, Madrid, Spain; ^8^ Clinical Practices of the Degree in Nursing, Universidad Francisco de Vitoria, Pozuelo de Alarcón, Spain

**Keywords:** non-communicable diseases, nursing, community health nursing, home nursing, house call, nursing interventions

## Abstract

**Objectives:** To explore nursing health education interventions for non-communicable disease patients.

**Methods:** The design was a systematic review of research work published between 2008 and 2018. The data sources included the Web of Science, PubMed, Scopus, COCHRANE, and LILACS. The studies that met the inclusion were assessed, and the analysis for methodological quality through the recommended tools CASPe, and JADAD.

**Results:** Fifteen original studies from eight counties were included in the review; Findings revealed 13 studies with randomized samples and six used power analysis. Nurses’ interventions included house calls, home care, and individual and group health education.

**Conclusion:** Nursing interventions showed 76.4% the effectiveness of results in patient outcomes to promote and improve healthier lifestyles and quality of life of non-communicable disease patients. This review discloses the significant impact of nursing health education interventions. Nursing leadership and political decision-makers should consider providing programs to enhance health education knowledge and abilities. All of this can favor the sustainability of the global economy by changing the life style of thousands of people worldwide.

**Systematic Review Registration:** https://www.crd.york.ac.uk/prospero/, identifier CRD42020208809.

## Introduction

The current increase in the prevalence and incidence of non-communicable diseases (NCDs), part of the chronic diseases realm, has become a priority for all health systems worldwide. The World Health Organization (WHO) has defined these conditions as being of long duration and generally slow progression and causing early death [1]. Cardiovascular diseases affecting the heart, several kinds of cancer, chronic respiratory conditions, diabetes, and rheumatic and neurological disorders are among the most frequent NCDs affecting all adult population [2, 3].

The NCDs have a significant impact on the population’s quality of life. It affects patients, families, and their environment with consequences for society, economic, psychological, and social levels [4]. Health systems in most counties need to undergo structural changes to provide healthcare services focusing on continuous surveillance and efficiency. It also should include investment in a specialized workforce and resources to guarantee the quality of care and an association with a network of auxiliary services.

These concerns require more research to provide new evidence and therapeutic advances, enhance institutional integration, and community health services, and close personalized and specialized family continuous surveillance. Improvements should favor distress among professionals [5, 6].

Within this framework, primary health care (PHC) has been considered an essential service associated with better health outcomes for individuals who live with NCDs [7]. In the PHC settings, nurses specialize in public health, and community nursing and nurse practitioners perform control and prevention of NCDs [8], as mentioned by Toney-Butler and Thayer 2019 [9]; Semachew 2018 [10]; Hanlon et al. 2018 [11]; Costa et al. 2016 [12]: Nursing services in PHC integrated into the different levels of the health system, with close interaction with hospitals and other agencies, should be an essential instrument in advancing NCD care.

Public health, community, or family specialized nurses should have the knowledge and abilities to identify problems and plan, implement, and evaluate quality nursing care. Their contribution to the control of NCDs could add to attaining goals to improve the quality of life of NDCs patients [13, 14], Gathering research evidence on the knowledge underlying nursing care should improve and reinforce their contribution to nursing science and practice [13, 14], while answering the population’s need for wellbeing and health [15, 16]. The tested relevance of nursing interventions and the outcome for the patients is indeed an imposition facing 21st century nursing [17].

Nursing interventions and teamwork cooperation with other healthcare professionals help to establish holistic, integrative healthcare. In this respect, nursing has shown extensive knowledge, skills, and attitudes, underlining their teamwork competencies as highly valued for necessary and systematic coordination within the multidisciplinary health team. In summary, nursing services should rely on specialized nurses at all levels of the health system [18].

In this order of ideas, studies conducted on nursing interventions within NCD show improvement in glycemic control [19], enhancement of quality of life [20], decrease in hospitalizations to complications, and effective reduction in mortality, as mentioned by Zegers et al. 2016 [21] and Lloyd et al. 2012 [22]. Evidence suggests that nursing services could be a vital instrument to help control healthcare costs, as mentioned by Laurant et al. 2018 [23]; Coleman et al. 2009 [24] and Cramm et al. 2013 [25].

Research has also provided control through restructured guidelines to improve the nursing practice and create a patient self-care culture effectively and efficiently. In addition, health administrators and managers in charge of developing policies have taken nurse-led NCD management programs as strategies to decrease the economic and healthcare burden of NCD. A review conducted in 2016 for Khanassov et al [26], shows that interventions organized and led by nurses improve the accessibility, availability, and affordability of primary healthcare services, improving health outcomes for the population. However, understanding and classifying these interventions at the international level can help the national health system learn from others and achieve the expected population health outcomes.

Nursing organizations’ leadership throughout the world since 2013, when the WHO Global Action Plan for the Prevention and Control of NCDs 2013–2020 was created, has pursued calling attention to the important role their professionals should assume in contribution to targets related to the reduction of premature mortality and improvement of life quality from NCDs [6]. In addition, in the realm of the UN Sustainable Development, nursing research centered on interventions of education and promotion of health to NDC patients and their families will certainly generate critical new knowledge to enhance the actions toward the attainment of these goals, good health and well-being, as stated in goal number 3: “Ensuring healthy lives and promoting well-being to CDC patients and their families [27–29].” Thus, the implementation of research about nursing-specific care is among the activities that provide evidence in this respect.

Nursing literature is somewhat limited; however, it has provided evidence of actions oriented toward guidance for self-care and information on community resources as a target in support of actions needed attaining the Sustainable Development Goals. Therefore, this study’s primary purpose was to conduct a systematic literature review to explore nursing health education interventions to promote improvement of the health and quality of life of NCD patients.

## Methods

### Study Design

The steps guiding this review included formulation of the main question, criteria of inclusion, literature search, and data assessment using a systematic process for identification, evaluation, and synthesis of the existing scientific evidence analysis and conclusions [30–33]. Firstly, the guiding principles based on PICO components were used to develop the questions for this systematic review: 1) Are nurses implementing actions to improve self-care and increasing knowledge about aid support in the community? 2) What method of health education is provided to NCDs patients, families, and home care personnel, if involved? 3) What kinds of information or guidelines are available? 4) What was the population of the study? 5) Do the studies include patient outcomes? As an independent variable, NCD patient-focused nursing interventions are used.

### Search Strategy

As a first approach, the search included five health science databases: Web of Science, PubMed, Scopus, COCHRANE, and LILACS, to find publication data between 2008 and 2018 and accomplished in December 2018. A secondary search aiming to retrieve all available information on clinical studies from grey literature, Ph.D. dissertations in the Spanish Dissertations database (TESEO), and The National Library of Medicine Gateway, was completed in January 2019. Healthcare science (DeCS) and Medical Subject Headings (MeSH) used descriptors. The keywords were: nurse, non-communicable diseases, intervention nursing, house calls, community health nursing. The Boolean operators used were intersection (AND) to establish the logical operations between concepts and (OR) to retrieve papers where at least one of the arguments specified appears.

### Inclusion and Exclusion Criteria

The study used the following criteria to select the studies: 1) randomized and non-randomized clinical studies; 2) participants older than 18 years; 3) NCD diagnosis; 4) complete description of educational level, interventions related to prevention including health education process, therapeutic procedures after diagnose, rehabilitation, as applied according to NCD, promotion 5) covering field of primary health nursing care, including home care; 6) published between 2008 and 2018; and 7) written in Spanish or English. Exclusion included papers with interventions led by non-nursing healthcare professionals or op-eds and discussion papers.

### Selection Process and Quality Assessment

**FIGURE 1:**
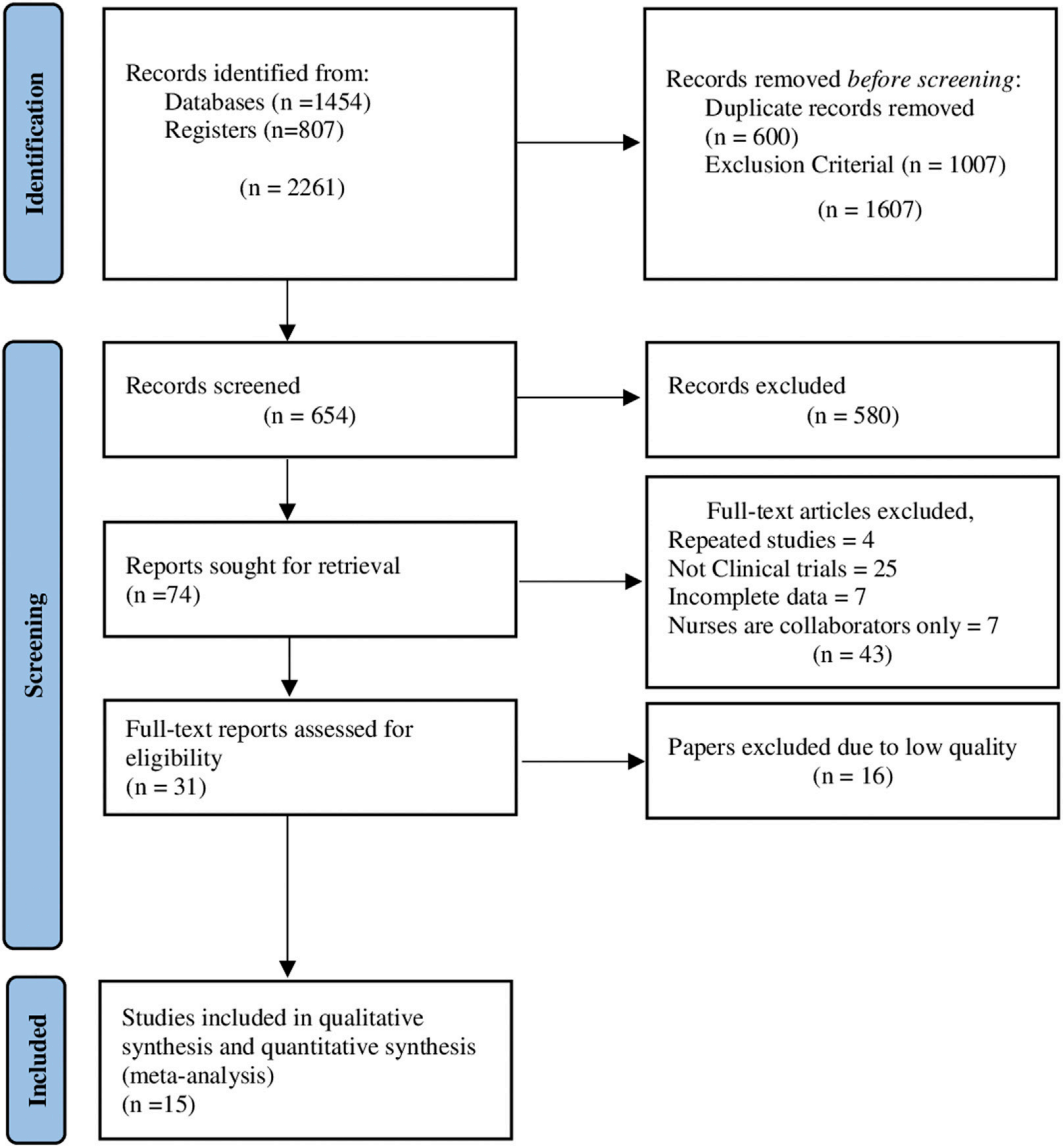
Flow diagram for systematic reviews (Page MJ, et al. The PRISMA 2020 statement: an updated guideline for reporting systematic reviews. 2021). [Sweden, The Netherlands, the UK, Colombia, Spain, Australia, Hong Kong, China, and Austria. (2009-2017)].

Figure 1 shows a PRISMA diagram summarizing the selection process. The results from the initial database search yielded 1,454 studies and the results from other sources (dissertations and grey literature) identified 807 papers, adding to a total of 2,261 papers; the screening process removed duplicates and studies not fulfilling the inclusion criteria (1607). A total of 74 abstracts remained for full-text screening, after the elimination of 580 articles that were independently reviewed by researchers where the intervention led by health professionals were not nurses or were opinion and discussion articles. In the process, 31 full‐text reports were assessed for eligibility and 16 papers were excluded due to low‐quality research methodology, and the remaining 15 papers were included in this literature review. The assessment of methodological quality used the CASPe [34] and JADAD tools [35].

The selected fifteen studies were independently peer-assessed and submitted to a test methodological minimum quality level [36]. Methodological quality was assessed through the CASPe [34] tool, which includes the validity, nature, and applicability of the results, and the JADAD scale [35] to evaluate the methodology of the studies and establish the extent of the possibility of bias in the design, conduct, and analysis.

The first author elaborated the abstraction and synthesis of the included studies that can be seen in Table 1, including aims and design, settings and sample, nursing CDs interventions, main findings, and quality evidence.

**TABLE 1:** Summary of Studies on Nursing Interventions for non-communicable diseases [Sweden, The Netherlands, the UK, Colombia, Spain, Australia, Hong Kong, China, and Austria. (2009-2017)].

Author (year) Country	Aim (s) and design	Setting and sample	Nursing CDs intervention	Main findings	Quality evidence
Ågren et al. [49] Sweden	To assess cost-effectiveness of a nurse-led health education and psychosocial support program to heart failures patients and their partners. Randomized dyads of patients and partner	Outpatient HF clinics and home Partners living in the same home. 155 patients 155 partners Patients IG (*n* = 71) CG (*n* = 84) Partners IG (*n* = 71) CG (*n* = 84)	IG - integrated nurse-led counselling, health education at home or at the HF clinic. The Intervention was delivered in three face-to-face sessions, computer base interchange, and written material. Held at two, six and 12 weeks after discharge, a counseling section focused on HF and the development of problem-solving skills. It also focused on thoughts and behaviors change, introducing and reinforcing strategies for self-care. CG - received standard care at the hospital and general health education and support at the outpatient HF clinic. Cost effectiveness included staff time, weights for patient and partner quality-adjusted of life-year using SF—6D.	One hundred and fifty-five dyads were included. Unitary cost per patient was €223. Both groups had positive quality of life enhancement at the end of 1 year. Significant difference was observed between the two groups. The Intervention was not regarded as profitable. However, positive outcomes observed in the IG dyads could represent a sufficient mean cost-effectiveness (not significant).	Moderate
Arts et al. [45] Netherlands	To evaluate the cost-effectiveness diabetes nurse as substitute of physicians thought the outcomes of intervention on clinical factors. Randomized non blind clinical trial.	Hospital in Maastricht. Diabetes Mellitus Specialized nurses (4) 285 patients IG: (*n* = 149) CG (*n* = 145)	Intervention group received care by four specialist nurses following a pre-set protocol. Control group received habitual attention provided by the five physicians. Costs, quality of life, and adverse events were measured, cost-effect ratios and incremental cost-effect ratios were calculated based on health-resource utilization rates, corresponding market prices, and national tariffs from 2007.	Independent t-test showed no statistically significant differences between mean EQ-5D scores for the two groups at baseline (p = 014). ANOVA did not show any statistically significant interaction effect between the nurse specialist or usual care (p-value = 0336) EQ-5D scores remained similar over time in both groups, non-significant decrease (p-value = 0.0058). Nurse specialists give diabetes care similar to those provided by physicians in terms of quality of life and economic value.	Moderate
Billington [51] United Kingdom	To promote auto-care to increase well-being and reduce acute problems in COPD patients. Single-center, two-tails randomized trial.	Primary health care COPD IG: (*n* = 35) CG: (*n* = 38)	Standard Intervention was the same for IG and CG for 6 weeks. To act in the IG and develop a baseline a nurse-led educational telephone intervention is performed. Additionally, an advanced nurse practitioner contacted twice; during the remaining weeks, a scheduled telephone appointment (an average of 25 min) to interchange information and counseling reinforcement systematically designed to guide and improve COPD self-management.	Follow-up CAT data were available for 69 of the 73 randomized patients. CAT scores in the IG decrease significantly, displaying an improvement between time 1 and 2 (Time 1 = 15.56 vs 12.44 in time 2, median Difference: 3.12. CI 1.52 –4.72, p-value < 0.05). A significant difference between the CAT scores of the IG and CG after adjustment for CAT baseline scores at time 1 (Mean difference = ––2.38, 95% CI –4.40 to – 0.36, p-value = 0.021). The Intervention did not cause additional costs.	High
Bohórquez et al. [42] Colombia	To determine the efficacy of the nursing interventions “Nursing fatigue of the caregiver role"(Nanda 00061), to provide support to the primary caregivers. Controlled clinical trial.	Hospital Universitario de Santander in Colombia—outpatient clinic and home visits Caregivers 30 IG: (*n* = 10) CG: (*n* = 20)	Each participant in the IG receives five training sessions on “support of the caregiver.” In addition, two home visits for two consecutive weeks (an average of 80 min) followed by three sessions of 2 h to accomplish 12 activities included the NIC out of a total of the 31.	The covariance analysis for the intervention result for the final NOC was 0.5 (p-value = 0.000, IC 95% 0.368; 0.626), indicating statistical significance; age was the supposed confounding variable. After adjustment for this variable, there was no significant change from the previous result. The NNT was 1 (IC 95% 1.00; 1.16)	High
Brotons et al. [46] Spain	To assess whether a home-based intervention reduces patients with heart failure, reduces mortality and hospital readmissions, and improves life quality. Randomized clinical study.	Home-based and hospital interventions Heart failure IG: (*n* = 144) CG: (*n* = 139)	Patients in the IG receive a formal orientation before discharge and home visits every month for 1 year aiming to reinforce and advise patients about their health condition and treatment regimen; the nurses also maintained a telephone contact every 15 days to check on their evolution. Patients CG returned to their GP or specialist and a citation to return to the hospital after 1 year.	The main hazard risk (HR) for each group was 41,7% for patients in the IG and 54,3% in the CG; HR of 0.70 (CI del 95%, 0.55–0.99). The HR lowered lightly with time (HR = 0.62; IC 95%, 0.50–0.87). At the final trial, the IG group reported a better quality of life (18.57 versus 31.11; p-value < 0.001). Mortality reduction and fewer rehospitalization.	Moderate
Brännsträm and Boman [50] Sweden	To evaluate the outcomes of PREFER model integrating specialized care for HF and palliative care. Prospective randomized study.	Department of Medicine-Geriatrics and Healthcare Centers 72 CHF patients IG: (*n* = 36) CG: (*n* = 36)	Usual care was provided mainly by general practitioners or nurses, the heart failure clinics at the. Regarding patient symptoms, health-related quality of life (HQRL), and hospitalizations compared with usual care	Analysis inter-group revealed that patients receiving PREFER had improved HRQL compared with controls (57.6 ± 19.2 vs 48.5 ± 24.4, age-adjusted p-value = 0.05). Intra-group analysis revealed a 26% improvement in the PREFER group for HRQL (p = 0.046) compared with 3% (p = 0.82) in the control group. Nausea was improved in the PREFER group (2.4 ± 2.7 vs 1.7 ± 1.7, p = 0.02), and total symptom burden, self-efficacy, and quality of life improved by 18% (p = 0.035), 17% (p = 0.041), and 24% (p = 0.047), respectively. NYHA class improved in 11 of the 28 (39%) PREFER patients compared with 3 out of the 29 (10%) control patients (p = 0.015). Fifteen rehospitalizations (103 days) occurred in the PREFER group, compared with 53 (305 days) in the control group. Potential to improve QoL in chronic heart failure patients.	High

Carrington and Stewart [37] Australia	To develop a cost-effective nursing program nurse-led to care to prevent the development of chronic heart failure. The mean purposes are: (i) to establish a clear healthcare plan using an expert guideline, and (ii) to establish a personal management regime to optimize the treatment of CVD; and (iii) to assure clinical stability, appropriate management, and risk profile. Randomized controlled trial.	Hospital, Outpatient, Home visit IG: (*n* = 375) CG: (*n* = 375)	CHF-MPs intervention is coordinated and run by a qualified specialist nurse in cardiac care and advanced training in the management of CVD and diabetes. Home visit for 18 months aiming to develop short and long-term strategies to prevent CHF development.	The primary (composite) endpoint of the NIL-CHF Study is event-free survival from a CHF-related hospitalization or all-cause mortality during 3–5 years of follow-up. Endpoints will be adjudicated by an independent and blinded Study End-Point Committee. Secondary endpoints will also examine the potential of the intervention to make a positive impact. Hospital costs for all reasons were also significantly lower (14%) in the intervention group of the trial ($AU 823 vs $ AU 960 per patient/year; p = 0.0045)	High
Chan et al. [44] Hong Kong	To determine whether an additional multi-component health education intervention increases the pneumococcal vaccination uptake rate among older patients with chronic diseases. Cluster randomized clinical trial.	Outpatient, Clinic IG: (*n* = 1251) CG: (*n* = 1266)	IG received brief health education guidance through telephone interchange before the 3-min face-to-face health education session during clinic visits. The content of interchange included healthcare and health problems on patient condition. CG received a reminder on their upcoming medical appointment after completing the baseline questionnaire.	The vaccination rate was higher in the intervention group compared to the control group (57% vs. 48%: relative risk = 1.20, 95% CI = 1.06–1.37). The two groups did not differ significantly in their awareness of the vaccination at 3-month follow-up (65% vs 59%, relative risk = 0.86, 95% CI = 0.69–1.07). 1.20 HR associated with the Intervention (CI 95% = 1.06–1.37). Intervention effectively improves pneumococcal vaccination of elder CDs patients No double-blind evidence	Moderate
Pardavila-Belio et al. [47] Spain	To evaluate the effectiveness of a nurse intervention aiming at helping college student smokers quit smoking. Single-blind, pragmatic randomized controlled	Where??? Prevention Nursing students level IG: (*n* = 133) CG: (*n* = 122)	It implemented a multi-component intervention based on the Theory of Triadic Influence of Flay. A nurse-let Intervention, including a 50-min motivational interview and online self-supporting and follow-up, including reinforcing e-mail and group therapy.	At the 6 month follow-up, the smoking cessation incidence was 21.1% in the IG than 6.6% in the CG (difference = 14.5 CI = 6.1–22.8; RR = 3.41, 95% CI = 1.62–7.20). The mean number of cigarettes at 6 months was significantly different (difference = –2.2, CI = –3.6 to – 0.9). Effective to increase smoking cessation.	High
Rojas-Sánchez et al. [43] Colombia	Evaluate nursing interventions for IMTR of CD patients through nursing home visits focusing on enhancing the knowledge about specific pathology and therapeutic measures. Controlled test.	IG: (*n* = 15) CG: (*n* = 30)	IG: Five home visits in addition to routine care between January and February of 2008. Integrative Instructional activities focused on the NIC’s five interventions according to the individual participant diagnoses. CG: Routine care	Final ANCOVA adjusted for NOC shows an average difference of 1.1 between the two groups. 1.1 (IC 95%: 0.6–1.6; p-value = 0.000) an increase of 1.5 (IC 95%: 1.0–2.0; p-value = 0.000) in the final score of each of the NOCs tags.	High
Soto et al. [48] Spain	To assess whether an educational intervention for perimenopause women with hypertension, diabetes mellitus, or dyslipidemia could significantly reduce biochemical and hemodynamic risk parameters. Randomized clinical test	Clinic Perimenopause women IG: (*n* = 160) CG: (*n* = 160)	The IG received three educational sessions covering the target content. Hemodynamic and biochemical variables were evaluated at baseline and 1 year later in both groups. The CG received handout information by mail.	The IG showed a decrease in low-density lipoproteins (p-value = 0.013) (2.71 ± 10.6; CI 95% −13.1/0.27), an increase in high-density lipoproteins (p-value = 0.013) (2.71 ± 10.6; CI 95%: −1.36/6.20), improved SBP (p-value value = 0.016) (−2.16 ± 11.8; CI 95%: −4.4/0.01), heart rate (p-value = 0.003) (−1.46 ± 10.3; CI 95%: −3.34/0.42) compared to women in the control group.	High
Stewart et al. [38] Australia	To examine the benefits of the same model of care nurse-led, multidisciplinary, HBI to prevent secondary events in hospitalized patients extended the continuum of heart disease. Randomized clinical trials (3)	IG: (*n* = 612) CG: (*n* = 614)	IG: Post-discharge, elements of the HBI were as follows: 1) a home visit from 7 to 14 days; 2) comprehensive automated reports derived from baseline; 3) coordination of multidisciplinary follow-up; 4) telephone follow-up; 5) a strong focus on the 6 months post-index hospitalization to address the residual risk of hospital readmission; and 6) structured review and generation of a comprehensive report and recommendations for future actions.	The IG achieved significantly lengthy free survival (90.1% CI: 95%, 88.2–92.0). Lower mortality (Adjusted risk, 0.67, CI 95%, 0.50–0.88; p-value = 0.005).	High
Stewart et al. [39] Australia	To determine the effectiveness of a long-term, nurse-led, multidisciplinary program of home/clinic visits to prevent progressive cardiac dysfunction and De Novo Chronic Heart Failure (CHF). Randomized control test	IG: (*n* = 310) CG: (*n* = 314)	IG received a multidisciplinary management program designed to prevent progressive cardiac dysfunction during 3–5.5 years follow-up There were two nursing support phases: (1) Home visits at the 7th to 14th day after discharge to reinforce the treatment regime, another visit at 1 month to further NIL-CHF clinic data. Subsequent management to adjust according to the Green Amber Red Delineation of risk and Need (GUARDIAN) tool. CG standard care	The intervention group displayed better cardiac recovery in the echocardiography after 3 years [81/226 (35.8%) vs 56/225 (24.9%), OR 1.44, CI 95% 1.08–1.92, p-value = 0.011].	High
Wang et al. [41] China	To test the effect of a Health Belief Model-based nursing intervention on healthcare outcomes in Chinese patients with moderate to severe COPD. A randomized controlled test	Hospital, Outpatient COPD IG: (*n* = 42) CG: (*n* = 46)	IG and CG received routine nursing standard care at the ward, covering HBM nursing education at the bedside. IG patients received health education guidelines 2 days after their disease conditions were stable. An average of two interventions held for IG before discharge. Based on the HBM. Additionally, patients in IG were taught how to prevent difficulties in breathing and maintain physical strength and continue oxygen therapy to guarantee that they could conduct the proper procedure when they were ready for it.	Results showed that the FEV1/FVC ratio’s value had a significant difference between study groups before and after the Intervention. Results also indicated that mean scores of the Dyspnea Scale, 6-min walking distance, and ADL was significantly different between the groups and between the study time-points. ANOVA, between-groups difference, patients with nursing intervention displayed greater benefits in their respiratory state than other traditional care combinations. (p-value < 0.01.).	Moderate
Zwar et al. [40] Australia	To assess the effectiveness of early Intervention of a team nurse-GP on quality of life (QoL) and the process of care in patients with newly diagnosed COPD, compared with usual care. A multicenter cluster randomized controlled trial.	Outpatient COPD IG: (*n* = 126) CG: (*n* = 90)	GPs and Nurses received previous training in team-based management of COPD. Content covered mainly pathophysiology and assessment of COPD; smoking cessation; evidence-based COPD management: inhaler technique; pulmonary rehabilitation; management of exacerbations; behavior change, teamwork, and fostering partnerships	QoL (ANOVA = -0.21 p-value = 0.86), influenza inoculation (OR 2.31: p = 0.035)	High

$AU, Australian Dollar; ANCOVA, analysis of covariance; ANOVA, analysis of variance; CAT, COPD assessment test; CG, control group; CHF, congestive heart failure; CI, confident interval; COPD, chronic obstructive pulmonary disease; NOC, nursing outcomes classification; CVD, cardiovascular disease; EQ-6D, EuroQol—% Dimension; FEV1/FVC, the ratio FEV1/FVC a value less than 70% indicates airflow limitation and the possibility of COPD; FEV1: volume that has been exhaled at the end of the first second of forced expiration; FVC, forced vital capacity; HBI, home-based intervention HBM, health belief model; HF, heart failure; HQRL, health related quality of life; HR, hazard risk; IG, intervention group; IMRT, intensive modulated radiation therapy; NANDA, North American nursing diagnosis association; NIC, nursing intervention classification; NNT, number need to treat; SBP, systolic blood pressure; PREFER, palliative advanced home caRE and heart FailurE care.

## Results

### Characteristics of Selected Studies

The fifteen studies were from eight countries [Australia [4] Carrington and Stewart 2009 [**37**], Stewart et al. 2016 [38], Stewart et al. 2015 [39]; Zwar et al. 2011 [40]; China [1] Wang et al. 2013 [41]; Colombia [2] Bohórquez et al. 2011 [42], Rojas-Sánchez et al. 2009 [43]; Hong Kong [1] Chan et al. 2015 [44]; Netherland [1] Arts et al. 2011 [45]; Spain [3] Brotons et al. 2009 [46], Pardavila-Belio el al. 2015 [47], Soto et al. 2017 [48]; Sweden [2] Ågren et al. 2013 [49], Brännström & Boman 2014(50); United Kingdom [1] Billington et al. 2015 [51]]. In thirteen studies, participants were reported as randomized and divided into two groups - intervention and control. Two studies were controlled clinical trials (Bohórquez et al. 2011 [42] & Rojas-Sánchez et al. 2009 [43]).

The sample size ranged between 30 and 2,517 (median:121). The median calculated was 58.8 ± 325.4 (range 20–1251) patients and 56.05 ± 328.9 (range 10–1266) for the intervention and control groups, respectively. Most studies reported their abandonment rates.

Six clinical studies used power analysis (Arts et al. 2012(45); Chan et al. 2015 [44]; Zwar et al. 2016 [40]; Billington et al. 2015 [51]; Stewart et al. 2015; Stewart et al. 2016 [40]). Nurses were the principal researcher (*n* = 8) (Pardavila-Belio et al. 2015 [47]; Zwar et al. 2016 [40]; Rojas-Sánchez et al. 2009 [43]; Bohórquez et al. 2011 [42]; Chan et al. 2015 [44]; Ågren et al. 2013 [49]; Brännström 2014(50); Arts et al. 2012(45)), five do not specify who did the recruitment (Brotons, et al [46], Carrington & Stewart [37], Stewart et al [39], Stewart et al [38], Wang et al [41]) and in two clinical studies subjects were recruited by another professional within the research group, as mentioned for Soto Rodríguez et al [48]. and Billington et al [51].

The studies covered several topics related to the interventions led by nurses in the care of patients with chronic diseases. Interventions most cited were: house call and home care, *n* = 8 (53.3%) (Bohórquez et al [42], Brotons et al [46], Rojas-Sánchez et al [43], Ågren et al [49]. Wang et al [41]. Billington et al [51]. Stewart et al [38]. Brännström and Boman [50]), health education activities (individual and group), *n* = 3 (20%) (Chan et al [44]. Soto-Rodríguez et al [48]. Zwar et al [39] = , and additional nursing intervention focusing on general nursing care was also observed *n* = 3 (20%) [18, 37, 45]. One study performed 3 linked trials testing nurse-led, multidisciplinary, and home-based intervention, using three different clinical managements *n* = 1 (6.7%) [38].

It is important to note that even with repeated interventions with the same topic, the methodologies and experimental designs used were different. For this reason, outcomes were heterogeneous and not comparable.

Most interventions included a framework for preventing complications caused by NCDs, such as decreasing the risk of developing heart failure, in Carrington and Stewart [37], Stewart et al [38], and Zwar, et al [40], Wang, et al [41]. However, some studies were more specific, improving quality of life [37, 39–42, 44, 45, 51], reducing symptoms caused by the disease, reducing biochemical and hemodynamic parameters, smoking cessation, preventing hospitalization, and effectively managing the therapy system. Just one study assessed the cost-effectiveness of replacing doctors with primary care nurses. Regarding the description of NCDs targeted by interventions, most were chronic obstructive pulmonary disease (COPD) 4 (27%) and heart failure (HF) 4 (27%).

### Metrics Outcome

Most studies yielded positive outcomes in one or more metrics. The interventions were conducted to increase knowledge or improve clinical data in the experimental group, including increasing patients’ quality of life (Arts et al [45], Bilington et al [51], Bohorquez, et al [42]; Carrington and Stewart [37], Chan, et al [44]; Stewart et al [39]. Wang, et al [41]; and Zwar, et al. 2011 [40]). Nursing interventions were effective and 76.4% of the research presented positive effectiveness results.

Due to the challenging situation of NCDs in the world, nursing interventions in house calls can positively affect the evolution and management of the disease, preventing various complications. Most studies extensively and carefully described the criteria for selecting subjects, as well as the effectiveness of the interventions (Agren et al. 2013; Arts et al. 2011; Bilington et al. 2015; Brotons, et al. 2009; Brännström and Boman. 2014; Chan, et al. 2015; Pardavila Belio, et al. 2015; Rojas-Sanchez, et al. 2009; Soto, et al. 2017; and Stewart et al. 2016), and even in those results that were not statistically significant, the intervention uptake was low and had no additional beneficial effect over usual care or participants’ health-related QoL [39, 40].

The study by Ågren et al [49], analyzed the intervention costs in institutions and organizations, specifically for NCD patients. It demonstrated success stories, where treatments were compared between doctors and nurses, which proved similar care from both professionals, providing similar economic value and quality of life. This similarity gives rise to an opportunity to save on healthcare costs, given that nurse labor cost is remarkably lower than physicians [23] (Coleman et al [24], Cramm et al [25]). However, interventions are not regarded as profitable for the patient alone, as they are an economic burden. However, it showed positive effects on the quality of life of patients undergoing the intervention [45].

## Discussion

This literature review was an attempt to answer two central questions–the kind of nursing interventions and main NCDs in which nurses intervene–to ensure best results as a path to identify scientific evidence of the effectiveness of these interventions, a significant challenge that discloses the gap between nursing theory and practice, as mentioned by Stetler [16] and DiCenso [15].

All studies used several types of clinical studies, including randomized controlled studies, controlled clinical studies, control groups, cost-benefit analysis, and a variety of mixed nursing interventions led by nurses, from formal health education, counselling, information interchange, briefings to psychological support, all related to the Nursing Interventions Classification (NIC), code 5,510 definition and activities listed as health education.

Health education is an essential competence for professional nurses working all health levels and settings, and indeed requires that health professions, especially nurses, know the learning process and teaching skills. Almost all the reviewed studies did not assess or mention the kind of education methodology used. Only three studies referred to a specific component of the educational intervention [1]; Pardavila-Belio et al [47]., who refer to interventions focusing on the Triadic Theory Influence, a theory that considers health behaviors in preventive interventions (Flay & Petraitis 1994(47) mentioned in Frank J. Snyder 2012) [2], Rojas-Sánchez et al [43]., focused on five interventions proposed by the NIC according to the individual participant diagnoses, and [3] Stewart et al. (2015) used the tool surveillance the Green Amber Red Delineation of Risk And Need (GUARDIAN) as a management tool to assess risk and need control frequency and intensity of future interventions [37, 41]; in addition, they used “The Health Belief Model,” an internationally validated model not thoroughly explored in Chinese patients with COPD.

Of the fifteen selected studies, 60% incorporated interventions that could include nursing care plans, for either the prevention, recovery, rehabilitation, or promotion of health. These interventions consisted of standardized care or the enforcement of protocol plans. These protocol plans are studies using multi-disciplinary interventions, motivational interviews, and other strategies. Forty percent of the remaining studies were part of the educational interventions group, including educational plans, follow-up telephonic interventions, and educational sessions. For this purpose, specialist professionals were chosen, accompanied by psychosocial programs or regular care. All studies observed that the intervention group (IG) and control group (CG) received routine health education care in the clinical setting.

The content of all interventions covered issues related to specific patient conditions [1] heart failure—6 (Agren et al.; Brännström and Boman; Brotons et al.; Carrington and Stewart; Stewart et al.; Stewart et al) [2]; COPD patients—3 (Billington et al.; Wang et al.; Zwar et al.) [3]; diabetes mellitus and other related disorders - 2 (Soto et al. and Arts et al.); The remaining studies did not focus on a specific pathology, but a condition related to the prevention of respiratory CDs 2 (Chan et al.; Pardavila-Belio el al.); IMRT to improve the adherence of CDs patients to treatment 1 (Carolina et al.) and, “fatigue” of primary care of patients with CDs—1 (Rojas-Sánchez et al.).

Despite differences in the procedures to measure the nursing interventions, the majority of the studies reviewed point out a significant change in patient lifestyle and their health condition control. This signifies the effectiveness of the educational interventions in the context of CDs specific conditions to improve the quality of life, support primary caregiver, acceptance of immunization program to prevent respiratory diseases, adherence to therapeutic treatment and smoking cessation program, decrease of risk factors, improved cardiac conditions in chronic heart failure, decrease mortality and rehospitalization, improvement of patients with moderate and severe COPD [38–41, 43, 44, 46, 47, 52, 53].

Three studies can be classified as a cost-effectiveness study. Two are of inferior quality due to the specific statement declaration as single-blind studies. In one of them, there is no evidence of the analysis of the results contemplating the group losses.

The three studies measured interventions guided by nurses about conventional care. Nonetheless, the results contemplate different units of analysis. These are total hospital cost for the intervened patients versus the control group, cost of intervention per patient and reduction of expenses in the direct cost of patient care. These results cannot be compared. In two of the three studies, the interventions were considered more cost-effective as compared to the control group. These interventions were displacing doctors for specialist nurses for the checking of patients with non-communicable diseases [45] and a program of care guided by a nurse for patients with chronic heart failure [37]. Another intervention was not regarded as profitable [49]. The third showed that the intervention was significant to improve health conditions of COPD patients and did not cause additional cost [51].

The present review also displays some evidence related to specialized and advanced practice nurses or nurses intervening in the educational process, or training sessions for nurses before the intervention. It is well known that the relevance nursing training programs at the university level emphasize the educational role of nursing in health education, and this has been supported and enforced by governments, nursing, and international organizations. The WHO and the ICN also emphasize the importance of nursing in the control of NCDs, mainly in the primary health care setting with a critical surveillance program, including coordinate and multi-professional care [6, 54]. NCDs are a real threat to human health. Over 50% of all causes of death in the world are chronic diseases, including the population in all counties, high and low income, young and elderly [55].

The study also mentions the role of nurses in support of non-professional care, these need information, training, as well as warmth and attention to their physical and psychological care [52], and strategies for home care, as mention by Brotons et al [46]. This illustrates the importance of home care, showing that it reduces mortality rates, and re-hospitalizations due to complications, and improves quality of life in patients with heart failure.

It is important to note that the search was carried out at the end of 2018, the year in which the Nursing Now movement began. However, a posteriori information search was added, which, is supported by more evidence that home monitoring of older patients with NCDs decreases hospital admission and demonstrably prevents falls and autonomy capacity deficits, according to the results found by Lui M, et al [56]., in 2021.

In addition, Sorensen A, et al [57]., argue through a clinical trial that the home visit favors medication control, and decreases the number of admissions or readmissions in health institutions, a fact that can aggravate the mental state of patients. For its part, the COVID-19 pandemic came to demonstrate how the monitoring carried out by nursing reduced infections and improved sanitary isolation in some cases, greatly increasing the health status of the population.

Taking into consideration the diversity of methodology, and necessary procedures to measure the proposed nursing intervention in all clinical studies in this literature review, it was impossible to perform a meta-analysis.

### Relevance for the Fulfillment of the Sustainable Development Goals

This review discloses the significant impact of nursing educational interventions for patients with different NCD conditions. It shows the actual trend of an above-average increase in the incidence and prevalence of NCDs worldwide. Nursing is called on to play a leadership role to create and promote innovative solutions to improve the quality and efficacy of the health system. Furthermore, this guarantees a healthy life and promotes well-being at all ages, especially in the population with NCDs. Therefore, nursing leadership should mainly promote and emphasize the training of nurses to assume a key and active role in primary care settings, emphasizing the development of competencies and skills to care for patients with NCDs [54]. All this can favor the sustainability of the global economy by changing thousands of people in the world; such is the example of the presence of the current epidemiological state.

This key role should include competencies and skills for cooperative work with healthcare professionals and stakeholders in society. It certainly involves rigorous and robust teaching of educational strategies focused on the importance of behavioral changes because of many interventions that deal with patients with NCDs. To do this, the nursing professional helps to achieve significant advances in life expectancy and reduce the risk of infection or complications in patients who most deserve care.

Nursing organizations need to provide standardization of interventions for NCD problems at the local level, and procedures and guidelines that fit the population in their context. Patients with NCDs need to acquire the confidence to deal with their process and seek help before the aggravation of their health status.

Another significant nursing contribution to NCD surveillance and nursing care in the health system is interventions focusing on health promotion and disease prevention. Unhealthy lifestyles, such as smoking, passive attitude, inadequate nutrition, and life stress, aggravate NCDs. Environmental and genetic conditions also play a role in the onset of NCDs that require interventions to promote lifestyle changes [58]. Indeed, nursing can contribute to the battle against these factors, and policies are required in the health system to support and improve the commitment of the nursing profession to the care of NCDs, as well as a social and comprehensive commitment to improve outcomes and the lifestyle of the population it serves. Thus, empowering nurses to play a vital role in NCD control would positively contribute to achieving this goal.

## Conclusion

The results of this review show how nursing has published results of research on several topics in the last ten years, with a comprehensive view of research studies, conducting clinical studies, where the independent variable was education and integral intervention focusing on patients with NCDs and generating a culture of active and effective involvement.

The growing international evidence suggests that interventions carried out by community nurses contribute to the favorable modification of risk factors, lifestyles, and control of NCDs.

The outcomes of the review are consistent, despite including various types of nursing interventions based on education and the subjects’ active involvement. They are useful in terms of preventing complications caused by diseases, decreasing the risk of developing heart failure, improving quality of life, reducing the symptoms caused by disease, improving biochemical and hemodynamic parameters, smoke cessation, preventing hospitalization, and effectively managing the therapeutic regime, as well as decreasing healthcare costs in the care of patients with non-communicable diseases.

Nursing interventions were effective in the analysis. 76.4% of the research had positive outcomes regarding the effects of the studies, which is a strong argument to support nurses’ work.
